# Optimal pricing and carbon emission reduction decisions for a prefabricated building closed-loop supply chain under a carbon cap-and-trade regulation and government subsidies

**DOI:** 10.1371/journal.pone.0287684

**Published:** 2023-06-29

**Authors:** Xuefang Sun, Yuyang Wang, Yuanyuan Li, Wenjing Zhu, Dehuan Yan, Jiahui Li

**Affiliations:** 1 School of Management Engineering, Qingdao University of Technology, Qingdao, China; 2 School of Civil Engineering, Qingdao University of Technology, Qingdao, China; Hebei Agricultural University, CHINA

## Abstract

This paper explores a two-level prefabricated building closed-loop supply chain (CLSC) comprising a retailer and a prefabricated building manufacturer (PBM) under carbon cap-and-trade legislation and the government subsidies of carbon emission reduction (CER). In this CLSC, the PBM and the retailer recycle used products through their independent recycling channels. The optimum pricing and CER strategies within both decentralized and centralized systems, respectively, are analyzed. The Stackelberg game is used in the decentralized system to determine the optimum PBM’s CER level and the retailer’s pricing. By analysis, it concludes that increasing the carbon trading price can stimulate prefabricated construction corporations to improve their CER level, and that the government subsidy rate has a great effect on the profits of the PBM. Numerical examples with sensitivity analysis are used to further evaluate the roles of important factors in the optimum CER and pricing solutions of the prefabricated building CLSC in two dissimilar systems.

## Introduction

With the acceleration of industrialization, global warming has surfaced human survival and development as one of the major dilemmas [1,2]. It is recognized that global warming can be alleviated by a sustained CERs [3]. In hopes of exploring a development model of economic with low-carbon, non-pollution, and sustainability, countries around the world have formulated several carbon laws and regulations, including carbon cap-and-trade regulation, and government subsidies for reducing emissions [4]. As an efficient approach to decline carbon emissions, the carbon cap-and-trade ordinance has achieved pretty good gains in various countries and regions at the moment [5]. In 2005, the European Commission began to implement greenhouse gas emission quotas administered for its member states, on which basis the European Emissions Trading System was created [6]. Eight carbon trading pilot programs have been founded in China since 2013, and a nationwide carbon trading platform was established in 2017 [7]. Furthermore, the government’s subsidized strategy for reducing carbon emissions has also developed into an indispensable motivation measure for reducing carbon emissions in New York, Tokyo, Japan, and London.

It can be seen from the data of the International Energy Agency, the construction business alone is related to 39% of global carbon dioxide emissions in 2019, with a continuation of the upward trend [8]. By 2050, the energy consumption of the construction industry will be twice that of 2010, and greenhouse gas emissions will increase by 50%-150% [9]. As one of the Four Major Carbon Emission Sectors (industry, construction, transportation, and electricity) defined by the Intergovernmental Panel on Climate Change, the construction industry nearly runs in opposite directions with the pressures of the low-carbon healthy development direction of society and economy. Accordingly, the task of lessening carbon releases in the construction business is urgent and required [10].

Fabricated buildings are commonly interpreted as buildings, with complete or semi-complete parts manufactured in the manufacturing plant and assembled at the construction scene [11]. As a part of the construction business, prefabricated constructions have benefits in environmental-protected, pollution reduction, energy saving, and efficiency, which have become the required way for all countries in the world to seek a low-carbon economy [12]. In 1991, the view that the growth of prefabricated buildings offered opportunities for the prosperity of the American construction industry was put forward at the PCI annual meeting held in the United States [13]. The Chinese government declares that by 2025, 30% of the nationwide new buildings every year will be prefabricated buildings [14]. Meanwhile, some prefabricated construction enterprises have sprung up around the world. Founded in 1912, Huf Haus is one of the most famous suppliers of prefabricated luxury villas in Germany. It has completed more than 10000 projects in the world so far. Since its establishment in 1960, Sekisui House’s business has spread to Japan, the United States, Australia, China, and the United Kingdom and has developed into an assembly giant in Japan. In this context, how to fulfill CER in the operation optimization of prefabricated buildings CLSC is a deserving area to be researched.

The correlation between government regulations and the actions of the prefabricated corporation has attracted the attention of several researchers [15–17]. Prominent decision-making enlightenment for the members of the prefabricated architecture supply chain can be provided effectively by their findings. Most of all, without affecting the sustainable economic growth of prefabricated building enterprises, reducing greenhouse gas emissions can be accomplished under the support of a carbon cap-and-trade legislation and government subsidies by setting mandatory carbon emission limits or providing relevant subsidies for prefabricated construction enterprises [18]. Firms can correctly adjust their production and operation decisions to maximize profits. These can be supported by this paper. In other words, CER level and price decision-making of the prefabricated construction corporation companies can be adjusted carefully under the influence of government regulations. However, in practice, two major challenges are faced by the operation of the carbon cap-and-trade legislation, and government subsidies for lightening emissions: how can the operational decisions of prefabricated construction companies be affected by the strict carbon cap-and-trade legislation and government subsidies for lightening emissions? How should the pricing, production, and operation decisions of prefabricated construction firms to adjusted with rigorous cap-and-trade regulations and government subsidies for lightening emissions?

To resolve these research-related difficulties, a prefabricated building CLSC, which comprises a retailer and a PBM, is considered under carbon cap-and-trade legislation and government subsidies. In this CLSC, production necessitates the release of carbon, and the retailer and the PBM recycle used products through their respective channels. Market demand is defined as a linear function that is influenced by the retailer’s pricing and the PBM’s CER standard. Then, decentralized and centralized models are developed for the perfect pricing and CER decision-making, respectively. For these two models, we derive the analytic expressions and provide the corresponding analytical solutions under carbon cap-and-trade legislation and government subsidies. Finally, numerical examples with sensitivity analysis are used to study the influences of certain key factors on the prefabricated building CLSC.

The leading contributions are threefold in this paper. Above all, we examine a two-echelon prefabricated building CLSC under government subsidies and carbon cap-and-trade legislation. In the decentralized system, we propose a Stackelberg game to measure the PBM’s CER standard and the retailer’s pricing strategies, in the centralized system, we provide a Hessian matrix. Second, closed-form expressions of the optimum CER standard and pricing solutions are obtained. Third, we investigated how optimum pricing and CER standard is shaped by carbon trading prices and government subsidy rates. The pricing is cheaper in the centralized system rather than in the decentralized system for the given carbon-trade price. Expanding the government subsidy rate can promote the CER standard.

The followings are the rest structure of this paper. We summarize the associated literature in Section 2. We describe the operating process of the prefabricated building CLSC with reasonable assumptions in Section 3. Under the decentralized model, the bearings of carbon regulation and subsidies of lessening discharge on the optimum pricing and CER standards of the prefabricated building CLSC are provided in Section 4. We present the optimum PBM’s CER standard and retailer’s pricing of the centralized model in Section 5. Section 6, complements the theoretical results by performing a numerical analysis. Section 7, conclusions and further discussion emerge.

## Literature review

In this section, we investigated the optimum CER and pricing strategies of a prefabricated building CLSC in a carbon cap-and-trade legislation and government subsidies. The literature reviewed involves three main research directions: (i) operational decisions with carbon cap-and-trade legislation; (ii) operational decisions with government subsidies; and (iii) prefabricated building supply chain models.

### Operational decisions with carbon cap-and-trade legislation

Many researchers have investigated the operational decisions of CLSCs under carbon cap-and-trade legislation.

Xu et al. [19] discovered that the mixed CLSC is emission-effective under carbon tax legislation and that a special CLSC is more cost-effective under carbon limit legislation. Mohammed et al. [20], under different carbon policies, analyzed supply chain tactics and pointed out that a carbon cap-and-trade policy is far more valid. In our paper, we further analyzed the impact of the carbon trading price on the optimal CER level and pricing of the CLSC. Li et al. [21] perceived the market demand depending on carbon discharge standards and pricing and considered the best strategies for a low-carbon CLSC. They found that, under the manufacturer-dominated system, cooperatively lessening carbon emissions improve used-product recycling and the degrees of CER. According to customers’ low-carbon preferences, Yang and Wang [22] based on the manufacturer’s remanufacturing capability and formed a model for diminishing carbon release in an ordinary supply chain with dual-channel.

Recently, Shu et al. [23] investigated the bearings of corporate social responsibility and carbon release restraints on a CLSC’s recycling and remanufacturing decisions. They discovered that the recycling rate directly correlates with the remanufacturing emission diminution coefficient. Zhang et al. [24] used Pontryagin’s maximum theory to cultivate the best dynamic retail price, total profit, and green innovation choice for a hybrid model with a carbon emission constraint. Under cap-and-trade legislation, Yang et al. [25] engrossed a remanufacturing CLSC, in which the gathering operations able to be changed by one third party, one retailer, or one manufacturer. They showed that the manufacturer’s optimal collection mode leads to more carbon emissions, in which the conditions are the lower carbon price or the higher carbon intensity. In simple manufacturing and flexible manufacturing-remanufacturing systems, the production and sustainability strategies under a carbon tax statute were addressed by Alegoz et al. [26]. Luo et al. [27] constructed four Stackelberg games to discuss the connection between a carbon tax and various fairness concerns of supply chain parts on the optimum strategy through the establishment of online direct channels. They found that the recycling decision of the manufacturer is not affected by fairly distributed attention to supply chain members but is affected by the carbon tax legislation, which represents as increasing the carbon tax can encourage the manufacturer to recycle more products.

### Operational decisions with government subsidies

To lower carbon releases, countries worldwide busily provide government subsidies to enterprises that take valid measures to control the level of carbon emissions. The role of government subsidy legislation in decision-making in the supply chain has been explored by quite a few researchers.

Zhou et al. [28] designed a two-echelon CLSC model under the condition of government recycling allowances to examine the change of the whole supply chain and carbon emission effects. Wan [29] developed a CLSC where the manufacturer obtains a low-carbon government subsidy for remanufactured products. From the perspectives of each member of the CLSC, they calculated the roles of the low-carbon allowance in the CLSC. The desirable recycling policy and pricing of a CLSC containing double reverse routes under two different subsidy policies were discussed by Wan and Hong [30]. Nielsen et al. [31] used different member-leading Stackelberg games to compare the outcomes of different allowance ordinances. The results in their model demonstrated that CLSC members can obtain more profits when a government allowance is provided directly to the consumer. Zhang et al. [32] built a Stackelberg dynamic model among the supply chain parts and government to look for the link between the CLSC’s decisions and reward-penalty ordinances.

In aspects of production mode, He et al. [33] ascertained the manufacturer’s perfect resolutions, they analyzed the optimum allowance standards for the government with each channel structure. Song et al. [34] considered the recovery of environmental sustainability products in a single-cycle CLSC under a government subsidy. Based on the Stackelberg game, they formulated three different donations to calculate the impact of subsidies on fresh product pricing and waste product recycling. In this paper, government subsidies for the PBM’s CER are introduced. Under the decentralized system, the optimum pricing and CER level is provided by using Stackelberg game, and we explore the corresponding optimal decisions of the prefabricated building CLSC under the centralized system. Presuming that the effects of reducing emissions, retail price, recycling, and advertising can change market demand, Shang et al. [35] constructed four different scenarios to search for the best strategies for a CLSC with the bearing of government interference.

### Prefabricated building supply chain models

In recent years, the trouble of contaminating circumstances and wasting resource has become increasingly serious in the traditional construction business. To overcome these troubles, prefabricated buildings are being broadly adopted worldwide and are attracting more attention from researchers, such as Li et al. [36], who wish to study the management of prefabricated building supply chains.

Han et al. [37] studied a prefabrication construction supply chain, including two-downstream contractors and one-component manufacturer, in which the larger contractor can produce the component by itself or purchase it through outsourcing and the smaller contractor obtains the component from the manufacturer. They built a Cournot-Stackelberg system to obtain optimal decisions on production, operations, and profit boundary conditions. Shi et al. [38] pointed out that properly combining subsidies with tax policies can encourage construction companies to apply prefabrication. Du et al. [39] structured many models to discuss the optimum assembly rate with different government subsidies and explored the optimum subsidy revenue-sharing coefficient and assembly rate under centralized and decentralized systems. They found that the optimum assembly rate is highest in the centralized system. Recently, Zhai et al. [40] studied buffer space hedging cooperation in the supply chain management of prefabricated architecture construction. Han et al. [41] studied the links among consumers, construction developers, and the government in the prefabricated construction domain. They got the optimum minimum assembly rate for government subsidy and equilibrium solutions for the construction developers’ optimum pricing and assembly rate.

Li et al. [42] incorporated a carbon pricing rule into a supply chain for prefabricated buildings to find the best unit carbon emissions, wholesale price, and retail price by considering price-dependent demand. Jiang and Qi [43] considered subsidies for prefabricated buildings and examined the avail of allowances on assemblers’ assembly rates and pricing. They observed that a subsidy ordinance can reduce the retail pricing of prefabrication architecture and improve the assembly rate. However, to obtain higher profits, the assembler is obliged to reasonably command the cost element. Based on game theory, Jiang et al. [44] tested the two-stage prefabricated structure supply chain possessing power structures and agile cap-and-trade and explored the best strategies under tariff contracts and dissimilar power structures. Taking advantage of a Stackelberg model, Jiang and Yuan [45] considered the collaboration in the supply chain of prefabricated buildings under the cap-and-trade ordinance.

The main focus of our proposed model is carbon cap-and-trade policy and government subsidies. To introduce the previous research in this realm, we conducted a literature survey on carbon cap-and-trade policy and government subsidies. Prefabricated buildings are the primary research background.

Table 1 shows the summary of previous studies in this realm and indicates that few studies were absorbed in prefabricated buildings under carbon cap-and-trade policy and government subsidies, especially in a prefabricated building CLSC. In the rest of this paper, we describe the contents of this paper in detail.

**Table 1:** Summary of the literature survey.

Author(s)	prefabricated building	Stackelberg game	Government subsidies	Carbon regulations		CLSC
				Carbon cap-and-trade policy	Carbon tax policy	
[36]	√					
[37]	√	√				
[38]	√		√		√	
[39]	√	√	√			
[40]	√	√				
[41]	√		√			
[42]	√				√	
[43]	√		√			
[44]	√	√		√		
[45]	√	√		√		
**This paper**	√	√	√	√		√

## Problem description and assumptions

In this section, we consider the most appropriate strategies for a two-echelon prefabricated building CLSC comprising one PBM and one retailer under cap-and-trade legislation and government subsidies. In the forward channel, based on the retailer’s quantity required, the PBM uses used products and raw materials to manufacture the final products and at the wholesale price *ω* wholesales the products to the retailer. Thereafter, the retailer at the retail price *p* retails the products to customers. In this process, as the primary mechanism for reducing carbon emissions, the PBM receives the limitation of a carbon cap-and-trade legislation that comprises a carbon cap *E* set by the government. When the unit product is produced, the unit carbon emission *e*_0_ occurs. Meanwhile, to support the PBM in reducing carbon emissions, subsidies for lessening carbon emissions at the CER subsidy rate *φ*_*m*_ are offered to the PBM by the government [46].

In reverse channel, used products from consumers are recycled by the PBM and the retailer through their channels at recycling prices *p*_0_ and *p*_1_, respectively. Collected from consumers, the used products are sold to the PBM by the retailer at a recycling price *p*_2_. The total recycling quantity is *D*_*R*_ The quantity of used products that the retailer directly recycled is *τD*_*R*_, where *τ* represents the loyalty level of the retailer to the recycled quantity and 0 ≤ *τ* ≤ 1. The quantity of used products directly recycled by the PBM is (1 − *τ*)*D*_*R*_.

**Fig 1:**
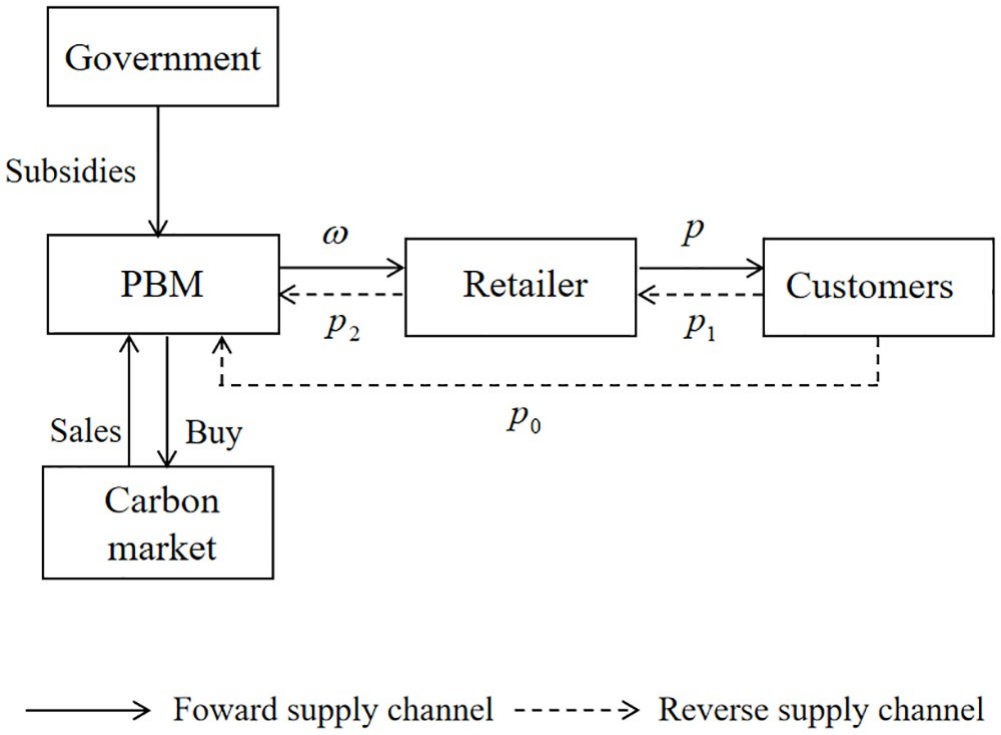
The two-echelon CLSC incorporate one retailer one PBM.

Under carbon cap-and-trade legislation and government subsidies, the PBM’s objective is to obtain the maximized profit by correctly setting the CER level *e*. And the retailer’s purpose is to maximize its profit by suitably deciding its pricing *p*. To analyze the perfect decisions of this CLSC under cap-and-trade legislation and government subsidies, the lot-for-lot principle is followed by the retailer, which is a typical presume in the relevant literature. The two-echelon CLSC that incorporate one retailer and one PBM is proved in Fig 1.

To develop the model, the assumptions adopted in this paper are as follows.

**Assumption 1.** The retailer and the PBM recycle used products through their channels, and no fierce race lies in them. The CER level decided the return quantity. The amount of recycled products grows as the CER level declines. We assume that *D*_*R*_ = *a*_1_ − *λ*_1*e*_, where *a*_1_ is a constant greater than zero that represents the fundamental recycled amount and *λ*_1_ is a sensitivity parameter for the degree of reduction in carbon emissions. The same assumption is adopted by Giri et al. [47].

**Assumption 2.** Following an assumption adopted by Savaskan et al. [48], there is no difference between products made of raw materials and made of used products.

**Assumption 3.** Let *D*(*p*,*e*) denote the retailer’s demand function. Based on the linear demand proposed by Huang et al. [49], and Xu et al. [50], the market demand is set as below: *D*(*p*,*e*) = *a* − *bp* + *λe*, where *α* is a constant and represents the retailer’s market potential, *b* is the price-sensitivity coefficient, *λ* is a low-carbon preference parameter of consumer and *a* > 0, *b* > 0, *λ* > 0, *a* > *ωb*.

**Assumption 4.** In a single-period setting, the CLSC decisions are taken into account.

**Assumption 5.** Some products previously sold can be recycled. The number of carbon emissions in the final products made from new materials and used products is equal. The unit cost of using used products to manufacture final products *c*_*r*_ is lower than that of using new materials to manufacture final products *c*_*n*_, and 0 < *c*_*r*_ < *c*_*n*_. To highlight the cost advantage of remanufacturing, we assume that *c*_*n*_ > *c*_*r*_ + *p*_0_ + *p*_2_. The same assumption is also adopted by Yuan et al. [51].

**Assumption 6.** The PBM invests in applying the low-carbon technique to decline carbon releases. Following Li et al. [21], we suppose that the cost of lessening carbon release *C*(*e*) is a convex function that is related to the CER standard *e* and is supposed to $C(e)=\frac{1}{2}Ue^{2}$, which *U* is a coefficient and refers to the CER effort cost.

**Assumption 7.** To obtain the closed-form solution, we adopt the assumption in Hua et al. [52] and assume there are no merchandising expenses involved in selling and recycling the products.

**Assumption 8.** To make sure the model’s accuracy, we assume in this paper that 2*bU*(*φ*_*m*_ − 1) < −(*λ* + *bp*_*c*_)^2^ < −4*λbp*_*c*_ < −4*λ*^2^.

The corresponding variables are marked with “*” to indicate their corresponding optimal values in the rest of this paper. In the decentralized mode, the relative variable is added with the subscript “1”; in the centralized system, the relative variable is added with the subscript “2”. To indicate the values of the retailer, the PBM and the total CLSC, this paper adds the corresponding subscripts “*r*”, “*m*”, and “*c*” to the necessary variables.

For convenient calculation, let *A* = *c*_*n*_ − *c*_*r*_ − (1 − *τ*)*p*_0_ − *τp*_2_, *B* = *c*_*n*_ − *c*_*r*_ − (1 − *τ*)*p*_0_ − *τp*_1_, *B*_1_ = *a* + *c_n_b* + *bp_c_e*_0_, *M* = *λ*_1_*A* − *λ*(*ω* − *c*_*n*_) + *p*_*c*_ (*λe*_0_ − *a*), *M*_1_ = *λc*_*n*_ + *λ*_1_*B* + *p*_*c*_ (*λe*_0_ − *a*), $N_{1}=\frac{1}{4b}{\left(a-\omega b\right)}^{2}+\tau (p_{2}-p_{1})a_{1}$, $N_{2}=Aa_{1}+\frac{1}{2}(a-\omega b)(\omega -c_{n})-p_{c}[\frac{e_{0}}{2}(a-\omega b)-E]$ and $N_{3}=\frac{1}{4b}{\left(a-c_{n}b-bp_{c}e_{0}\right)}^{2}+a_{1}B+p_{c}E$.

## Model analysis of the decentralized system

In the decentralized system, the decision-making procedure is established as a Stackelberg game where the follower is the retailer and the leader is the PBM. In this system, above all, the PBM determines the CER standard *e*. Afterward, the retail price *p* is chosen by the retailer with consideration of the PBM’s decision.

Following Jiang and Qi [53], the profit functions of both the PBM and retailer under this system can be designed as

$$\Pi_{m}(e)=(\omega -c_{n})(a-bp+\lambda e)+A(a_{1}-\lambda_{1}e)+\frac{Ue^{2}(\phi_{m}-1)}{2}-p_{c}[(e_{0}-e)(a-bp+\lambda e)-E],$$ (1)

$$\Pi_{r}(p)=(p-\omega )(a-bp+\lambda e)+\tau (p_{2}-p_{1})(a_{1}-\lambda_{1}e).$$ (2)

The PBM’s carbon releases are *D*(*p*,*e*)(*e*_0_ − *e*) right now.

From Eqs (1) and (2), we get the retailer’s optimum decision using the Stackelberg equilibrium theory. Then, the subsequent Lemma can be derived.

**Lemma 1.** The retailer’s profit is concave in *p* and can be designed as

$$\text{max}\;\Pi_{r}={\left(\frac{a+\lambda e-b\omega }{2b}\right)}^{2}+\tau (p_{2}-p_{1})(a_{1}-\lambda_{1}e).$$ (3)

We concluded the following realization for the PBM.

**Lemma 2.** The PBM’s profit is concave in *e*. The corresponding mathematical model of the PBM is

$$\begin{array}{c} \text{max}\;\Pi_{m}=(\omega -c_{n})\frac{U(\phi_{m}-1)(a-bp)+\lambda p_{c}(2a-bp)+\lambda M}{U(\phi_{m}-1)+2\lambda p_{c}}\\ +A\left[a_{1}-\frac{\lambda_{1}(M+bp_{c}p)}{U(\phi_{m}-1)+2\lambda p_{c}}\right]+\frac{U}{2}{\left[\frac{M+bp_{c}p}{U(\phi_{m}-1)+2\lambda p_{c}}\right]}^{2}(\phi_{m}-1)\\ -p_{c}\{\left[e_{0}-\frac{M+bp_{c}p}{U(\phi_{m}-1)+2\lambda p_{c}}\right]\frac{U(\phi_{m}-1)(a-bp)+\lambda p_{c}(2a-bp)+\lambda M}{U(\phi_{m}-1)+2\lambda p_{c}}-E\} \end{array}$$ (4)

We obtain the following conclusion from Lemmas 1 and 2.

**Theorem 1.** The optimal decisions under the decentralized system are

$$e_{1}{}^{*}=-\frac{2M+p_{c}(a+\omega b)}{2U(1-\phi_{m})-3\lambda p_{c}},$$ (5)

$$p_{1}{}^{*}=\frac{(a+\omega b)[U(1-\phi_{m})-2\lambda p_{c}]-\lambda M}{2bU(1-\phi_{m})-3\lambda bp_{c}}.$$ (6)

In Appendix A, a whole proof can be discovered.

**Theorem 2.** The optimal profits under the decentralized system are

$$\Pi_{r1}{}^{*}=\frac{\lambda^{2}}{4b}{\left[\frac{2M+p_{c}(a+\omega b)}{2U(\phi_{m}-1)+3\lambda p_{c}}\right]}^{2}+\frac{2M+p_{c}(a+\omega b)}{2U(\phi_{m}-1)+3\lambda p_{c}}[\frac{\lambda (a-\omega b)}{2b}+(A-B)\lambda_{1}]+N_{1},$$ (7)

$$\Pi_{m1}{}^{*}=-\frac{\lambda p_{c}}{4}{\left[\frac{2M+p_{c}(a+\omega b)}{2U(\phi_{m}-1)+3\lambda p_{c}}\right]}^{2}+\frac{2M+p_{c}(a+\omega b)}{4[2U(\phi_{m}-1)+3\lambda p_{c}]}[p_{c}\left(a-\omega b\right)-2A\lambda_{1}]+N_{2}.$$ (8)

In Appendix B, a whole proof can be discovered.

For optimal solutions of the retailer and the PBM in decentralized systems, we explore how the CER effort coefficient, carbon trading price, and government subsidy rate for reducing emissions influence the optimum solutions of the retailer and the PBM. We draw the following corollary.

**Corollary 1.** (1) $\frac{\partial p_{1}{}^{*}}{\partial U}<0,\frac{\partial p_{1}{}^{*}}{\partial \phi_{m}}>0,\frac{\partial p_{1}{}^{*}}{\partial p_{c}}>0.$ (2) $\frac{\partial e_{1}{}^{*}}{\partial \phi_{m}}>0,\frac{\partial e_{1}{}^{*}}{\partial p_{c}}>0,\frac{\partial e_{1}{}^{*}}{\partial b}<0.$

In Appendix C, a whole proof can be discovered.

Based on the above inferences, the PBM’s CER level improves to avoid increases in transaction cost, as the carbon trading price adds. To prevent profit loss, the PBM and the retailer add its price. When the government subsidy rate increases, the PBM can obtain additional profits due to adopting low-carbon technology. The PBM prepares to invest more in carbon release diminution for improving its CER level to win more profits. However, since the total cost adds, the PBM enhances the wholesale price to prevent the loss of profits, which causes the retailer’s retail price to increase.

In summary, the carrying out of carbon legislation and government subsidies will improve the PBM’s CER level. For this reason, cap-and-trade legislation and government subsidies for reducing emissions are conducive to the healthy development of prefabricated building enterprises.

**Corollary 2.** The PBM affordable minimal cap under the decentralized system is valid only when

$$E_{m1}\geq -\frac{\lambda }{2}{\left[\frac{2M+p_{c}(a+\omega b)}{2U(\phi_{m}-1)+3\lambda p_{c}}\right]}^{2}+\frac{\left(\lambda e_{0}-a+\omega b)[2M+p_{c}(a+\omega b)\right]}{2[2U(\phi_{m}-1)+3\lambda p_{c}]}+\frac{e_{0}(a-\omega b)}{2}.$$ (9)

In Appendix D, a whole proof can be discovered.

We can conclude that many factors affect the acceptability of the PBM’s minimum carbon cap from Corollary 2. If the carbon cap is higher than the PBM affordable minimal cap, the PBM sells the excess carbon cap in the carbon trading market for winning extra income. If the carbon cap is lower than the affordable minimal cap, to maximize profits, the PBM improves its CER standard, and purchases the cap from the carbon trading market, which results in the total cost increases. To reduce additional expenditure, the PBM strives to boost its CER level.

## Model analysis of the centralized system

In this section, the CER standard *e* and the retail price *p* are decided collectively by the retailer and the PBM. Inspired by Qi et al. [54], the profit of the prefabricated building CLSC is given as

$$\Pi_{c}(e,p)=(p-c_{n})(a-bp+\lambda e)+B(a_{1}-\lambda_{1}e)+\frac{Ue^{2}(\phi_{m}-1)}{2}-p_{c}[(e_{0}-e)(a-bp+\lambda e)-E].$$ (10)

In the centralized system, the PBM’s carbon releases are the total carbon releases now, that is *D*(*p*,*e*)(*e*_0_ − *e*).

**Lemma 3.** The CLSC’s profit is concave under the centralized model in *e* and *p*. The corresponding mathematical programming is

maxΠ(e,p)c (11)

We obtain the following conclusion from Lemma 3.

**Theorem 3**. The CLSC’s best strategies and the relevant profit in this system are

$$e_{2}{}^{*}=\frac{(\lambda +bp_{c})(a-c_{n}b-bp_{c}e_{0})-2\lambda_{1}bB}{2bU(1-\phi_{m})-{\left(\lambda +bp_{c}\right)}^{2}},$$ (12)

$$p_{2}{}^{*}=\frac{B_{1}[U(1-\phi_{m})-2\lambda p_{c}]-M_{1}(\lambda -bp_{c})}{2bU(1-\phi_{m})-{\left(\lambda +bp_{c}\right)}^{2}},$$ (13)

Π*c2=[−B1(λ−bpc)+2bM1](λ+bpc)(a−cnb−bpce0)2bU(ϕm−1)+(λ+bpc)2+N3. (14)

In Appendix E, a whole proof can be discovered.

To refine the CLSC’s best solutions in a centralized system, we explore how the coefficient of CER effort and the government subsidy rate affect the optimal solutions of the retailer and the PBM. We draw the following corollary.

**Corollary 3.** $\frac{\partial p_{2}{}^{*}}{\partial U}>0,\frac{\partial p_{2}{}^{*}}{\partial \phi_{m}}<0.$

In Appendix F, a whole proof can be discovered.

Based on the above inference, when the government subsidy rate adds, the CLSC adopting low-carbon technology can win more subsidies. Therefore, the total revenue of the CLSC has increased. As the CLSC’s total profit adds, the PBM and the retailer are willing to lower retail prices to attract more consumers. In general, the rise of the government subsidy rate is beneficial for reducing the retail price of prefabricated building CLSC and expanding the market share of prefabricated buildings.

**Corollary 4.** The PBM’s minimum carbon cap under the centralized system exist only when

$$E_{m2}\geq -\frac{\lambda +bp_{c}}{2}{\left[\frac{-B_{1}(\lambda -bp_{c})+2bM_{1}}{2bU(\phi_{m}-1)+{\left(\lambda +bp_{c}\right)}^{2}}\right]}^{2}+\frac{e_{0}(a-c_{n}b-bp_{c}e_{0})}{2}+\frac{\left(\lambda e_{0}-a+c_{n}b)[-B_{1}(\lambda -bp_{c})+2bM_{1}\right]}{2[2bU(\phi_{m}-1)+{\left(\lambda +bp_{c}\right)}^{2}]}.$$ (15)

In Appendix G, a whole proof can be discovered.

From Corollary 4, we know that many factors affect the acceptability of the PBM’s minimum carbon cap. The PBM sells the excess carbon cap in the carbon trading market for winning extra income if the carbon cap exceeded the affordable minimal carbon cap, to maximize profits, the PBM improves the CER standard. The PBM purchases the cap from the carbon trading market if the carbon cap is below the affordable minimal cap, to reduce additional expenditure, the PBM strives to boost its CER.

## Numerical experiments

In this section, the above analytical results are demonstrated by numerical analysis. Additionally, we study that the main parameters bear on the optimal pricing and CER of the prefabricated building CLSC.

### Example analysis

To demonstrate the above theoretical results in Sections 4 and 5, the following three numerical examples are used. The following are common parameters: *a* = 120, *b* = 0.8, *λ* = 0.1, *c*_*n*_ = 120, *c*_*r*_ = 40, *ω* = 140, *τ* = 0.6, *p*_1_ = 55, *p*_2_ = 70, *a*_1_ = 2, *λ*_1_ = 0.25, *φ*_*m*_ = 0.3, *U* = 120, *p*_*c*_ = 10, *e*_0_ = 2, and *E* = 3.

When *p*_0_ is 50, 65 and 70, and Table 2 shows the optimum strategies and relevant numerical results.

**Table 2:** Optimal decisions for three cases.

Optimal decisions	*p*_0_ = 50 *p*_2_ > *p*_1_ > *p*_0_		*p*_0_ = 65 *p*_2_ > *p*_0_ > *p*_1_		*p*_0_ = 70 *p*_0_ > *p*_2_ > *p*_1_	
	Decentralized system	Centralized system	Decentralized system	Centralized system	Decentralized system	Centralized system
*p*	145.0269	141.1241	145.0280	140.9518	145.0284	140.8944
*e*	0.4303	0.7850	0.4485	0.8199	0.4545	0.8315
*D*	4.0215	7.1792	4.0224	7.3205	4.0227	7.3676
*E*	6.3126	8.7228	6.2409	8.6391	6.2169	8.6090
Π_*r*_	37.2475	24.3037	37.2158	23.1230	37.2052	22.7186
Π_*m*_	73.5916	92.9431	62.2467	83.3275	58.4711	80.1447
Π_*c*_	110.8391	117.2468	99.4625	106.4505	95.6763	102.8634

From Table 2, we can conclude the following:

1. As the PBM’s recycling price increases, the market demand under the two systems expand. Simultaneously, under the same recycling price, the market demand is always greater in the centralized system. When *p*_0_ > *p*_2_ > *p*_1_, the greatest market demand of the retailer in the centralized mode is *D* = 7.3676. The minimum market demand of the retailer under the decentralized system is *D* = 4.0215 in the case of *p*_2_ > *p*_1_ > *p*_0_.
2. As the PBM’s recycling price adds, the CER level adds in the decentralized and centralized systems. Simultaneously, for a given recycling price, the CER standard is always greater in the centralized system. When *p*_0_ > *p*_2_ > *p*_1_, the highest CER standard in the centralized system is *e* = 0.8315. The lowest CER standard under the decentralized system is *e* = 0.4303 in the case of *p*_2_ > *p*_1_ > *p*_0_.
3. As the recycling price of the PBM adds, the PBM’s recycling cost adds, and the PBM’s profit dwindles in the two models. At the same time, under the same recycling price case, the PBM’s profit is invariably higher in the centralized system compared to the decentralized system. When *p*_2_ > *p*_1_ > *p*_0_, the CLSC wins the maximum profit in the decentralized system.

### Impacts of key parameters on the optimum decisions

**Fig 2:**
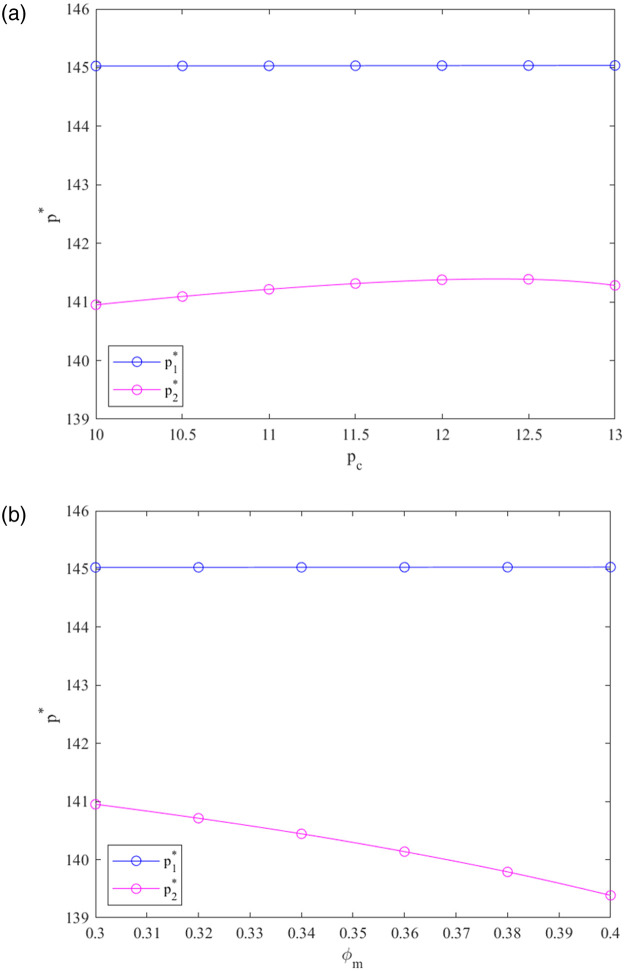
Effects of *p*_*c*_ and *φ*_*m*_ on p*.

We analyze the effects of critical factors on the best solutions of the prefabricated building CLSC in two systems. Fig 2 depicts the effects of *p*_*c*_ and *φ*_*m*_ on the retailer’s optimal pricing under the decentralized and centralized systems.

From Fig 2, the following can be seen:

1. The retailer’s optimum pricing is relatively stable when the carbon trading price *p*_*c*_ adds under the decentralized system. As *p*_*c*_ explodes, the retailer’s pricing in the centralized system decreases. Compared to the decentralized system, the optimum pricing of the retailer is lower in the centralized system under the given *p*_*c*_. That is conducive to the market demand expansion in the prefabricated building CLSC under the centralized system.
2. Compared with the corresponding decentralized system, the pricing is lower in the centralized system. With the growth of *φ*_*m*_, the retail price rapidly decreases in the centralized system and remains relatively stable in the decentralized system. The increase in the government subsidy rate *φ*_*m*_ promotes a decrease in retail price, which confirms the earlier theoretical findings in Corollaries 1 and 3.

**Fig 3:**
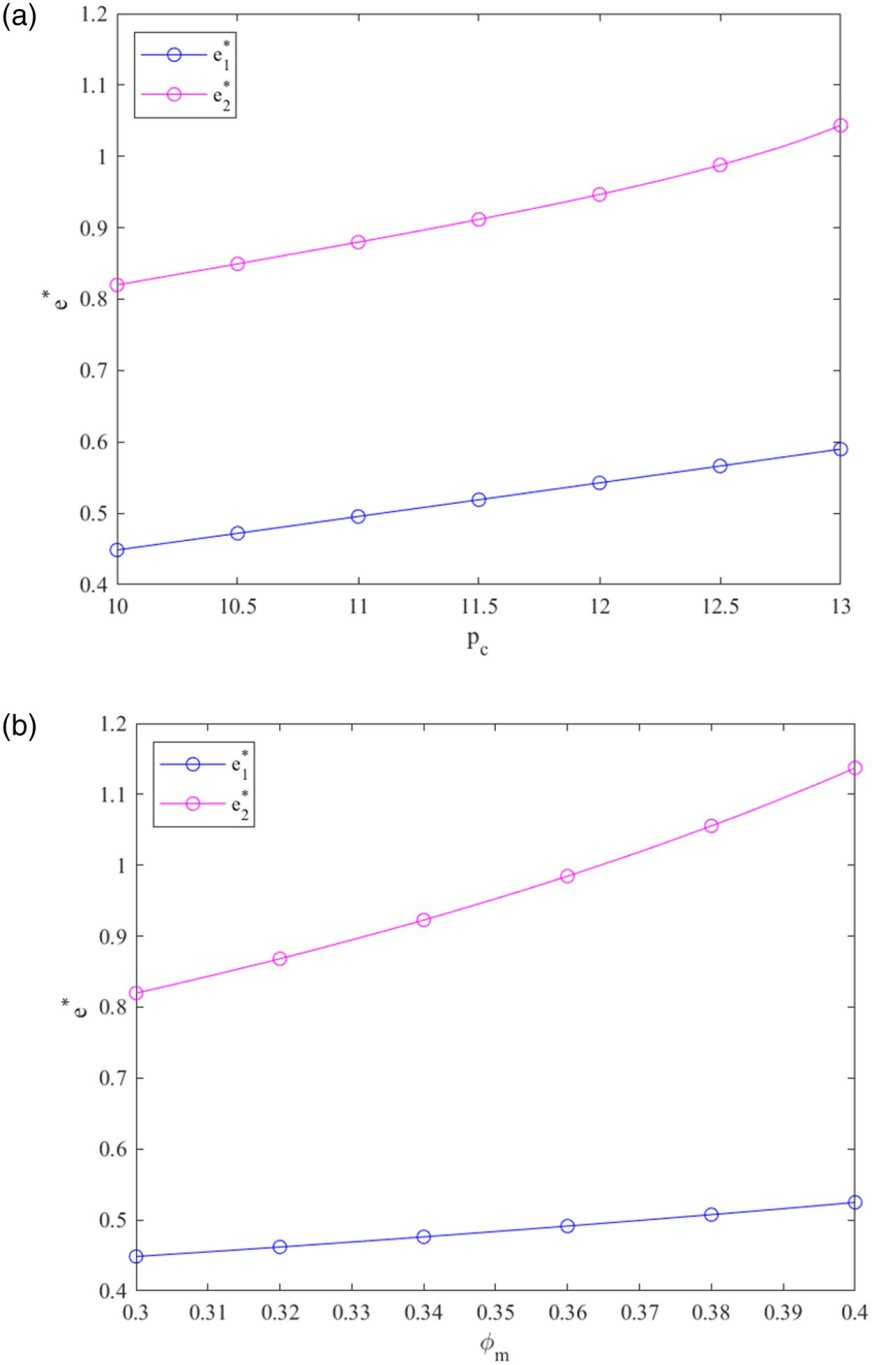
Effects of *p*_*c*_ and *φ*_*m*_ on e*.

From Fig 3, we can see the following:

1. Under decentralized and centralized decisions, the PBM’s optimum CER standard rises when the carbon trading price *p*_*c*_ adds. As *p*_*c*_ explodes, the PBM’s CER standard slowly adds in the decentralized system and adds relatively rapidly in the centralized system. Compared to the centralized system, the PBM’s optimum CER standard is lower in the decentralized system under the given *p*_*c*_. It can not be instrumental in the prefabricated building participants expanding low-carbon economics in the decentralized system.
2. Compared to the centralized system, the PBM’s CER standard is lower in the decentralized system. As *φ*_*m*_ growing, the PBM’s CER standard adds in the decentralized and centralized systems. The aforementioned findings in Corollary 1 are strengthened by the fact that the government allowance rate enhanced fosters the enhancement of the PBM’s CER standard in the two systems.

**Fig 4:**
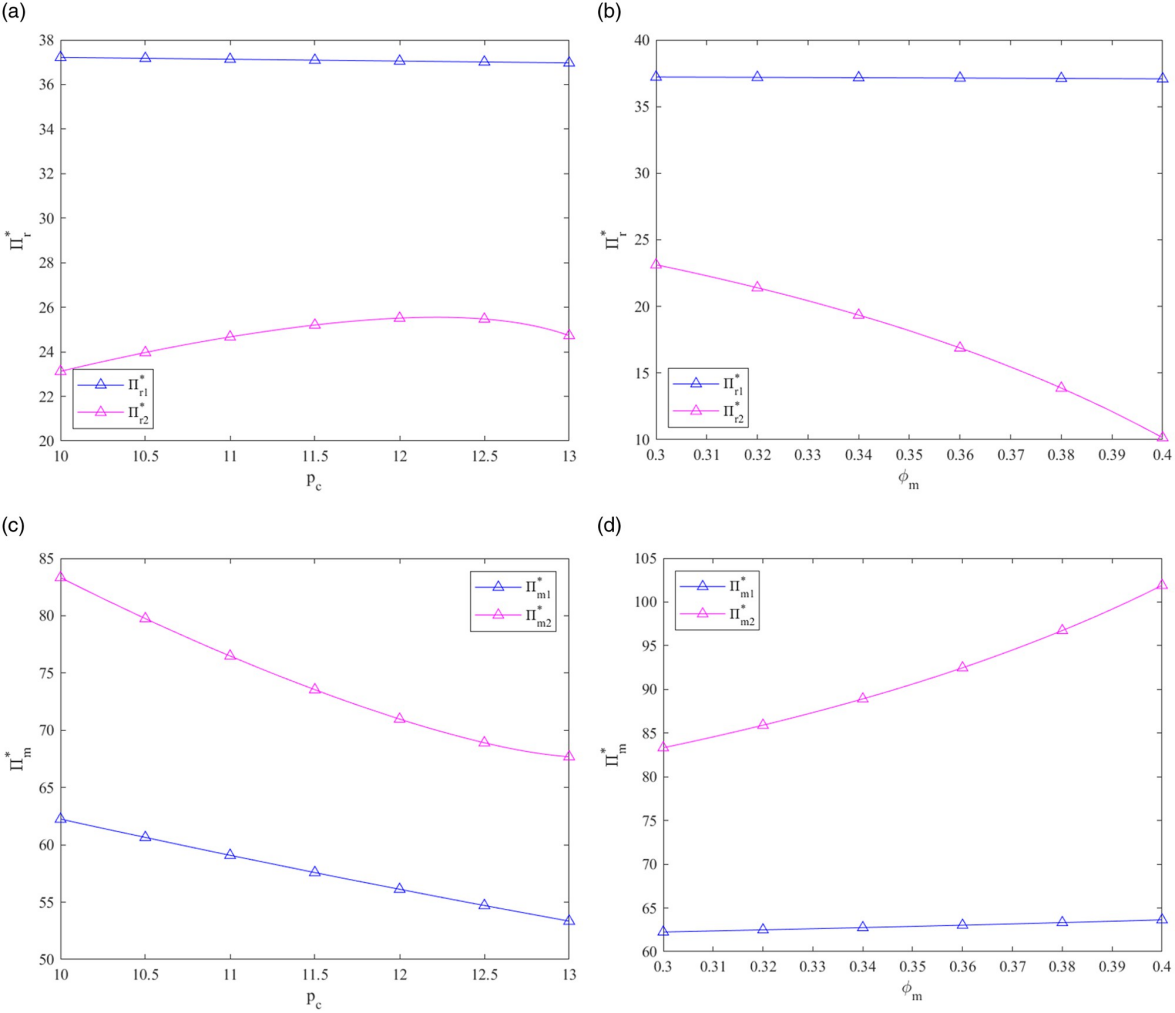
Effects of *p*_*c*_ and *φ*_*m*_ on Π_r_* and Π_m_*.

From Fig 4, we can see the following:

1. As *p*_*c*_ explodes in these two systems, the profits of the PBM are hurt. The retailer chooses the decentralized decision-making strategy, and the PBM chooses the centralized decision-making strategy. Additionally, when *p*_*c*_ < 10, the profit gap between prefabricated building CLSC members under decentralized and centralized systems may increase. At that time, choosing the way of decision-making counted for much meaning.
2. As the government subsidy rate *φ*_*m*_ explodes in the centralized system, the retailer’s earnings reduce, and the PBM’s earnings rise. The optimum earnings of every participant in the CLSC are relatively stable under the decentralized design when *φ*_*m*_ increases. The prefabricated building CLSC participants choose different models with the goal of maximizing profits, as *φ*_*m*_ changes.

**Fig 5:**
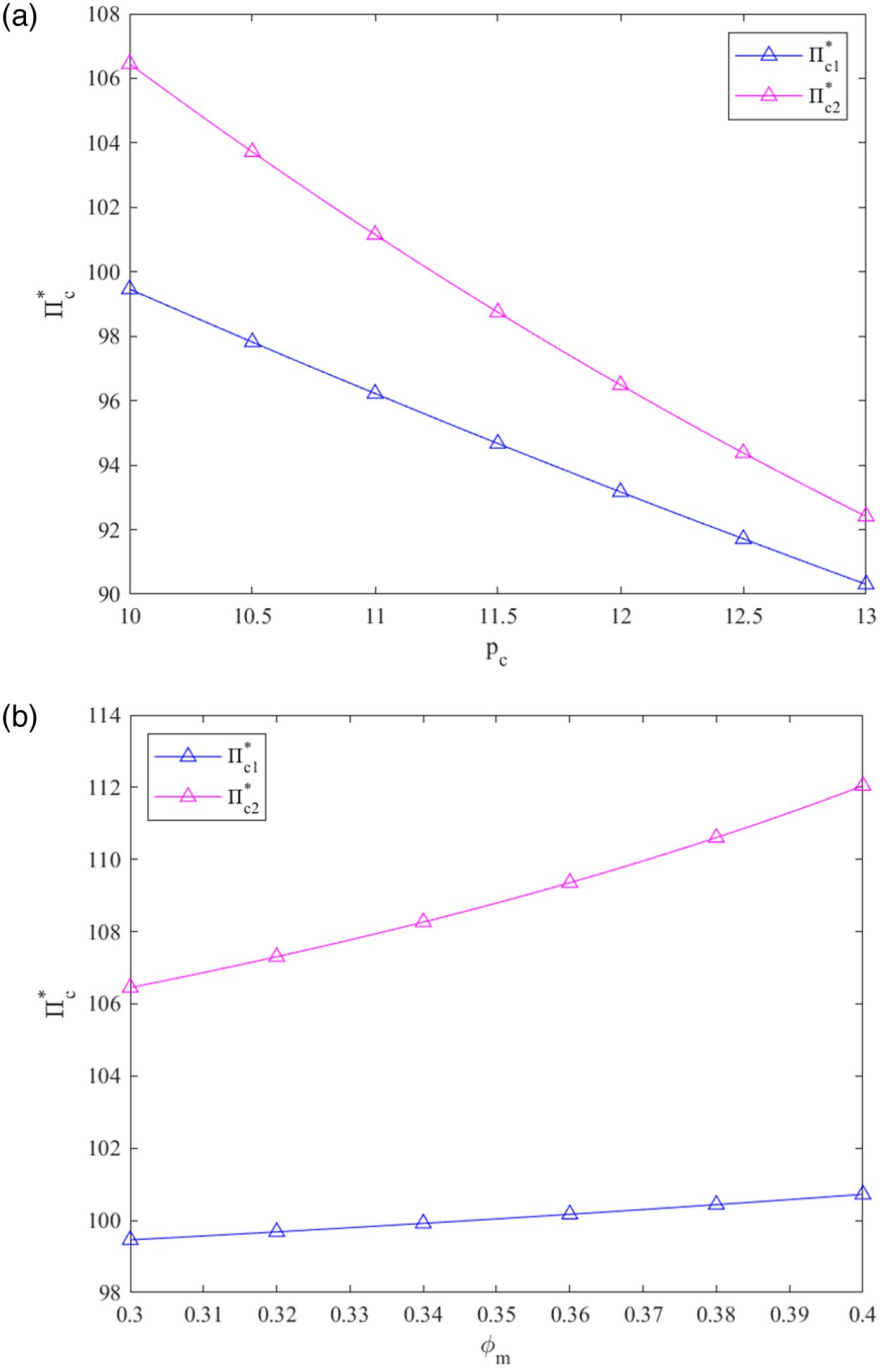
Effects of *p*_*c*_ and *φ*_*m*_ on Π_c_*.

From Fig 5, we can see the following:

1. The profit of each part in the CLSC decreases in these two systems as the carbon trading price *p*_*c*_ adds. For a given *p*_*c*_, the CLSC’s gain in most cases is better in the centralized strategy than in the decentralized strategy. Hence, it’s safe for each member of the CLSC to choose the centralized decision-making strategy.
2. As *φ*_*m*_ explodes, the CLSC’s gain rapidly adds in the centralized system and adds relatively slow in the decentralized system. The CLSC’s gain is higher in the centralized strategy than in the decentralized strategy under a given *φ*_*m*_. This is desirable for each member of the prefabricated building CLSC to select the centralized decision-making strategy.

## Conclusions

With the aggravation of environmental pollution in the construction industry, governments around the world have issued a series of carbon regulations. Under the influence of carbon regulations, the decision-making environment of construction enterprises has become more complex. Therefore, scholars and managers of prefabricated construction enterprises pay more attention to this issue. In this paper, a prefabricated building CLSC is considered, comprising one PBM and one retailer, against the background of carbon cap-and-trade legislation and government subsidies for CERs. The market demand in this prefabricated building CLSC is supposed to be a linear function with the retailer’s pricing and the PBM’s CER standard. The retailer and the PBM recycle used products through their respective independent recycling channels. Using the Stackelberg game, we test the optimum projects of the retailer’s pricing and the PBM’s CER standard in the decentralized system under carbon cap-and-trade legislation and CER subsidies. And then we extend the model into the centralized system and provide the optimum solutions through an analytical approach. Finally, we demonstrate the above-presented strategies through numerical examples and show sensitivity analysis to survey the roles of critical parameters on the optimum solutions of a prefabricated building CLSC.

### Managerial implications

This study has the following three leading management significance. First, when other influencing factors remain unchanged, the increase in carbon trading price can encourage enterprises to improve their CER level. In this case, for the sustainable development of enterprises and to avoid additional transaction costs, prefabricated construction enterprises invest more in CER costs, which is conducive to fostering a positive social image for enterprises. Second, the profit of the prefabricated building supply chain is closely related to the government subsidy rate. If the subsidy rate increases, this encourages prefabricated construction enterprises to strive to improve the CER level, which is conducive to attracting consumers with low carbon preference, expanding the demand for the prefabricated construction market, and increasing the profits of the prefabricated construction supply chain, and promoting the sound development of the construction industry. Finally, the rise of carbon trading price is beneficial to increase the retailers’ profits, while the increase of the government subsidy rate favors the prefabricated building manufacturers’ profits. Therefore, it counts for much to set goals and choose the correct decision-making mode when prefabricated building companies must make decisions in a complicated context.

### Limitations and future research

This paper considers the optimum pricing and CER decisions for a prefabricated building closed-loop supply chain under a carbon cap-and-trade policy and government subsidies. It enriches the correlative literature on the prefabricated building closed-loop supply chain. Extending our work in the following way will be interesting. It is assumed that there is no recycling competition between the PBM and the retailer in this paper. In reality, the recycling abilities of the PBM and the retailer interact with each other. Thus, by considering the competition between the PBM and the retailer, the present model can be developed. Additionally, the market demand in this paper is related to CER standards and pricing. However, the market demand can similarly be affected by other factors, such as assembly rate. This model can be developed by adding market demand influencing factors. Therefore, the author’s future research direction is to study the optimal decision-making of prefabricated building supply chain with recycling competition and assembly rate under carbon regulations.

## Supporting information

S1 File (DOC)
