# Supplementary material for: Optimal pricing and carbon emission reduction decisions for a prefabricated building closed-loop supply chain under a carbon cap-and-trade regulation and government subsidies
Source: PLoS One. 2023 Jun 29;18(6):e0287684. doi: 10.1371/journal.pone.0287684 (PMC10309996; doi:10.1371/journal.pone.0287684)
Supplement: S1 File — (DOC) [file pone.0287684.s001.doc]

**Supporting information**

**S1 Fig. 1. The two-echelon CLSC incorporate one retailer one PBM.**

**S2 Table 1. Optimal decisions for three cases.**

**S3 Fig. 2. Effects of $p_{c}$ and $\phi_{m}$ on the optimum retail price.**

**S4 Fig. 3. Effects of $p_{c}$ and $\phi_{m}$ on the optimum carbon emission reduction levels.**

**S5 Fig. 4. Effects of $p_{c}$ and $\phi_{m}$ on the optimum profit.**

**S6 Fig. 5. Effects of $p_{c}$ and $\phi_{m}$ on the CLSC’s profit.**

**Appendices**

**Appendix A. Proof of Theorem 1**

According to Eqs. (2) and (1), we have

$\frac{\partial\Pi_{r}(p)}{\partial p}=a-2bp+\lambda e+\omega b,$ (A.1)

$\frac{\partial^{2}\Pi_{r}(p)}{\partial p^{2}}=-2b<0,$ (A.2)

$\frac{\partial\Pi_{m}(e)}{\partial e}=\lambda(\omega-c_{n})-\lambda_{1}[c_{n}-c_{r}-\tau p_{2}-(1-\tau)p_{0}]+eU(\phi_{m}-1)-p_{c}(\lambda e_{0}-a+bp-2\lambda e),$ (A.3)

$\frac{\partial^{2}\Pi_{m}(e)}{\partial e^{2}}=U(\phi_{m}-1)+2\lambda p_{c}<0.$ (A.4)

$\Pi_{r}(p)$ is a concave function of $p$, and $\Pi_{m}(e)$ is a concave function of $e$. If we set $\frac{\partial\Pi_{r}(p)}{\partial p}=0$ and $\frac{\partial\Pi_{m}(e)}{\partial e}=0$, we can obtain the optimal solutions $e_{1}^{*}$ and $p_{1}^{*}$.

**Appendix B. Proof of Theorem 2**

If we substitute $e_{1}^{*}$and $p_{1}^{*}$ into Eqs. (2) and (1), we get $\Pi_{r1}^{*}$ and $\Pi_{m1}^{*}$.

$$\begin{matrix} \Pi_{r1}^{*} =(p^{*}-\omega)(a-bp^{*}+\lambda e^{*})+\tau(p_{2}-p_{1})(a_{1}-\lambda_{1}e^{*}) \\ =\frac{\lambda^{2}}{4b}[\frac{2M+p_{c}(a+\omega b)}{2U(\phi_{m}-1)+3\lambda p_{c}}]^{2}+\frac{2M+p_{c}(a+\omega b)}{2U(\phi_{m}-1)+3\lambda p_{c}}[\frac{\lambda}{2b}(a-\omega b)+(A-B)\lambda_{1}]+N_{1}, \end{matrix}$$

where $A=c_{n}-c_{r}-(1-\tau)p{}_{0}-\tau p_{2}$, $B=c_{n}-c_{r}-(1-\tau)p{}_{0}-\tau p_{1}$, $M=-\lambda(\omega-c_{n})+\lambda_{1}[c_{n}-c_{r}-\tau p_{2}-(1-\tau)p_{0}]+p_{c}(\lambda e_{0}-a)$, $N_{1}=\frac{(a-\omega b)^{2}}{4b}+\tau(p_{2}-p_{1})a_{1}$ and $N_{2}=\frac{(a-\omega b)(\omega-c_{n}-e_{0}p_{c})}{2}+Aa_{1}+p_{c}E$.

**Appendix C. Proof of Corollary 1**

As $e_{1}^{*}>0$, we can get $2\lambda(\omega-c_{n})-2\lambda_{1}A-p_{c}(2\lambda e_{0}-a+\omega b)>0$. Therefore,

$$\frac{\partial p_{1}^{*}}{\partial U}=\frac{\lambda bU(\phi_{m}-1)\{ap_{c}-\omega bp_{c}-2\lambda p_{c}e_{0}+2[\lambda(\omega-c_{n})-\lambda_{1}A]\}}{[2bU(\phi_{m}-1)+3\lambda bp_{c}]^{2}}<0,$$

$$\frac{\partial p_{1}^{*}}{\partial\phi_{m}}=\frac{\lambda bU\{ap_{c}-\omega bp_{c}-2\lambda p_{c}e_{0}+2[\lambda(\omega-c_{n})-\lambda_{1}A]\}}{[2bU(\phi_{m}-1)+3\lambda bp_{c}]^{2}}>0.$$

According to $e_{1}^{*}>0$, it can be get that $\lambda(\omega-c_{n})-\lambda_{1}A>\frac{1}{2}p_{c}(\omega b-a+2\lambda e_{0})$ and $2\lambda(\omega-c_{n})-p_{c}(\omega b-a+2\lambda e_{0})>2\lambda_{1}A$, Thus,

$\begin{matrix} \frac{\partial p_{1}^{*}}{\partial p_{c}}=\frac{\lambda bU(\phi_{m}-1)(\omega b-a+2\lambda e_{0})+3\lambda^{2}b[\lambda(\omega-c_{n})-\lambda_{1}A]}{[2bU(\phi_{m}-1)+3\lambda bp_{c}]^{2}} \\ >\frac{\lambda bU(\phi_{m}-1)(\omega b-a+2\lambda e_{0})+\frac{3}{2}\lambda^{2}bp_{c}(\omega b-a+2\lambda e_{0})}{[2bU(\phi_{m}-1)+3\lambda bp_{c}]^{2}}>0 \end{matrix}$, $\begin{matrix} \frac{\partial e_{1}^{*}}{\partial p_{c}}=\frac{2U(\phi_{m}-1)(\omega b-a+2\lambda e_{0})+6\lambda^{2}(\omega-c_{n})-6\lambda\lambda{}_{1}A}{[2bU(\phi_{m}-1)+3\lambda bp_{c}]^{2}} \\ >\frac{2U(\phi_{m}-1)(\omega b-a+2\lambda e_{0})+3\lambda p_{c}(\omega b-a+2\lambda e_{0})}{[2bU(\phi_{m}-1)+3\lambda bp_{c}]^{2}}>0 \end{matrix}$, $\frac{\partial e_{1}^{*}}{\partial\phi_{m}}=2U\frac{[2\lambda(\omega-c_{n})-p_{c}(\omega b-a+2\lambda e_{0})+2\lambda_{1}A]}{[2U(\phi_{m}-1)+3\lambda p_{c}]^{2}}>0$, $\frac{\partial e_{1}^{*}}{\partial b}=\frac{\omega p_{c}}{2U(\phi_{m}-1)+3\lambda p_{c}}<0$.

**Appendix D****. Proof of Corollary 2**

$$\begin{matrix} E_{m1}\geq(e_{0}-e)D \\ =(e_{0}-e)\frac{a+\lambda e-\omega b}{2} \\ =(e_{0}-e)(\frac{a-\omega b}{2}+\frac{\lambda}{2}e) \\ =-\frac{\lambda}{2}e^{2}+e(\frac{\lambda e_{0}}{2}-\frac{a-\omega b}{2})+\frac{e_{0}(a-\omega b)}{2} \\ =-\frac{\lambda}{2}[\frac{2M+p_{c}(a+\omega b)}{2U(\phi_{m}-1)+3\lambda p_{c}}]^{2}+\frac{\lambda e_{0}-a+\omega b}{2}\frac{2M+p_{c}(a+\omega b)}{2U(\phi_{m}-1)+3\lambda p_{c}}+\frac{e_{0}(a-\omega b)}{2}. \end{matrix}$$

**Appendix E. Proof of Theorem 3**

From Eq. (10), we have

$\frac{\partial\Pi_{c}(e,p)}{\partial e}=\lambda(p-c_{n})-\lambda_{1}[c_{n}-c_{r}-\tau p_{1}-(1-\tau)p{}_{0}]+U(\phi_{m}-1)e-p_{c}(\lambda e_{0}-a+bp-2\lambda e),$(E.1)

$\frac{\partial\Pi_{c}(e,p)}{\partial p}=a-2bp+\lambda e+c_{n}b+bp_{c}(e_{0}-e),$ (E.2)

$\frac{\partial^{2}\Pi_{c}(p)}{\partial p^{2}}=-2b<0,$ (E.3)

$\frac{\partial^{2}\Pi_{c}(e)}{\partial e^{2}}=U(\phi_{m}-1)+2\lambda p_{c}<0,$ (E.4)

$\frac{\partial^{2}\Pi_{c}(e,p)}{\partial p\partial e}=\frac{\partial^{2}\Pi_{c}(e,p)}{\partial e\partial p}=\lambda-bp_{c}.$ (E.5)

According to assumption 8, we can get $H(e,p)=\left|\begin{matrix} \frac{\partial^{2}\Pi_{c}(e,p)}{\partial e^{2}}\frac{\partial^{2}\Pi_{c}(e,p)}{\partial e\partial p} \\ \frac{\partial^{2}\Pi_{c}(e,p)}{\partial p\partial e}\frac{\partial^{2}\Pi_{c}(e,p)}{\partial p^{2}} \end{matrix}\right|=2bU(1-\phi_{m})-(\lambda+bp_{c})^{2}>0.$ Therefore, $\Pi_{c}(e,p)$ is a concave function of $e$ and $p$. Let $\frac{\partial\Pi_{c}(e,p)}{\partial e}=0$ and $\frac{\partial\Pi_{c}(e,p)}{\partial p}=0$, we get $e_{2}^{*}$ and $p_{2}^{*}$. If we substitute $e_{2}^{*}$ and $p_{2}^{*}$ into Eq. (10), $\Pi_{c2}^{*}$can be solved.

**Appendix F. Proof of Corollary 3**

As $\lambda<bp_{c}$, it can easily be proven that $\frac{\partial p_{2}^{*}}{\partial U}=\frac{(\phi_{m}-1)(\lambda-bp_{c})[(\lambda+bp_{c})(a-c_{n}b-bp_{c}e_{0})-2b\lambda_{1}B]}{[2bU(\phi_{m}-1)+(\lambda+bp_{c})^{2}]^{2}}>0$ and $\frac{\partial p_{2}^{*}}{\partial\phi_{m}}=\frac{U(\lambda-bp_{c})[(\lambda+bp_{c})(a-c_{n}b-bp_{c}e_{0})-2b\lambda_{1}B]}{[2bU(\phi_{m}-1)+(\lambda+bp_{c})^{2}]^{2}}<0$.

**Appendix G. Proof of Corollary 4**

$$\begin{matrix} E_{m2}\geq(e_{0}-e)D \\ =(e_{0}-e)\frac{a-c_{n}b-bp_{c}e_{0}+e(\lambda+bp_{c})}{2} \\ =(e_{0}-e)[\frac{a-c_{n}b-bp_{c}e_{0}}{2}+\frac{(\lambda+bp_{c})}{2}e] \\ =-\frac{\lambda+bp_{c}}{2}e^{2}+e[\frac{e_{0}(\lambda+bp_{c})}{2}-\frac{a-c_{n}b-bp_{c}e_{0}}{2}]+\frac{e_{0}(a-c_{n}b-bp_{c}e_{0})}{2} \\ =-\frac{\lambda+bp_{c}}{2}e^{2}+\frac{\lambda e_{0}-a+c_{n}b}{2}e+\frac{e_{0}(a-c_{n}b-bp_{c}e_{0})}{2} \\ =-\frac{\lambda+bp_{c}}{2}[\frac{-B_{1}(\lambda-bp_{c})+2bM_{1}}{2bU(\phi_{m}-1)+(\lambda+bp_{c})^{2}}]^{2}+\frac{(\lambda e_{0}-a+c_{n}b)[-B_{1}(\lambda-bp_{c})+2bM_{1}]}{2[2bU(\phi_{m}-1)+(\lambda+bp_{c})^{2}]}+\frac{e_{0}(a-c_{n}b-bp_{c}e_{0})}{2}. \end{matrix}$$
